# Supplementary material for: Compacting and correcting Trinity and Oases RNA-Seq de novo assemblies
Source: PeerJ. 2017 Feb 16;5:e2988. doi: 10.7717/peerj.2988 (PMC5316280; doi:10.7717/peerj.2988)

**Contig validation using exon re-alignment and order checking.**

The idea is to compare the assembled contig sets by comparing their exon content with the reference transcriptome. Two elements are verified, the exon presence and order.

The reference transcriptome is made of all the expressed transcripts (above nil expression level).


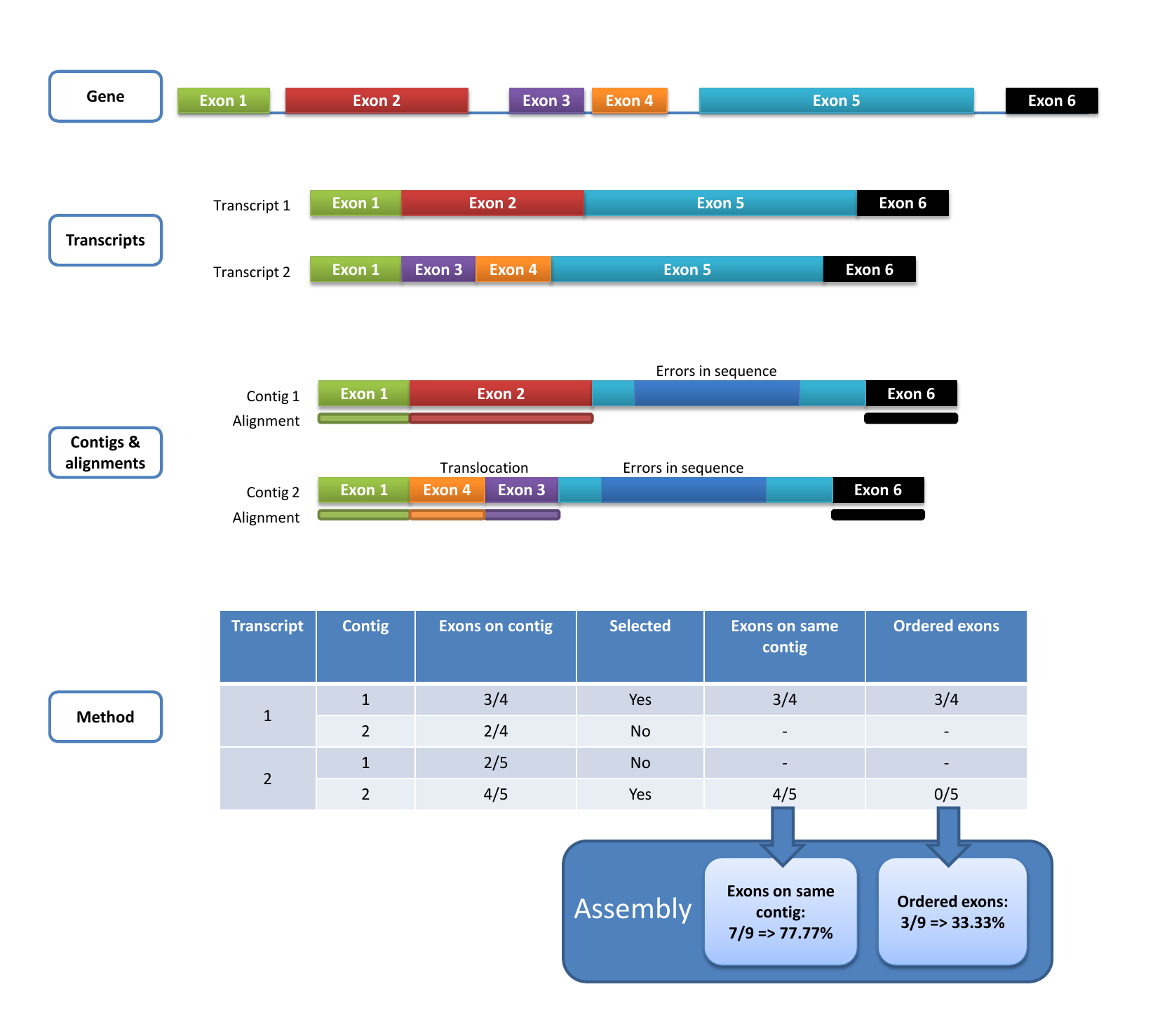
Exon presence is checked by aligning the exons of the reference transcripts on the contig set using BLAST (filtered by 80% identity and 80% of coverage). Using the number of hits, each transcript is linked to the corresponding contig: the contig with the highest number of corresponding exons is retained for each transcript. Then the order of the exons on the contig is verified and only the exons of contigs having all their exons respecting the initial order are counted. Gaps in the exon order are not taken into account. Several transcripts and genes can be linked to the same contig. The final figures are calculated for all the genes. The diagram hereunder presents assembly examples with only one gene and two transcripts which have been assembled in two contigs.

Fig. S1. Steps of the evaluation process and final marks

The diagram at the top of the figure presents the references gene structure with its exon order. The figure above shows the two corresponding transcript structures. The contig and alignment section present the contigs linked to the transcripts. The first one comprises an exon (exon 5) which does not align on the reference and the second includes a translocation of exon 3 and 4 and the same mis-alignment of exon 5. The table at the bottom presents how the contigs are linked to the transcripts by counting the number of aligned exon (the link is presented in the selected column) and then the mark given to each contig taking into account the correctness of the exon order in the contig.

The final marks of the assembly will be first the number of correctly reconstructed exons divide by the number of exons of the reference transcripts and second number of correctly ordered and reconstructed exons divide by the number of exons of the reference transcripts.

**Contig set correction step assessment.**

In order to verify the impact of the contig correction step, the reference proteins have been aligned to the simulated dataset (Ds) DRAP contigs before and after correction using BLAT. The score evolution of the best hit for each DRAP contig has been observed. The table hereunder presents the number of hits between reference proteins and DRAP contigs impacted by the correction step.

| **Dataset** | **Assembler** | **Nb contigs** | **Nb contigs with hit** | **Nb hits with impacted score** | **Nb hits positively impacted** | **Nb hits negatively impacted** | **Positive impact (%)** | **Negative impact (%)** |
| --- | --- | --- | --- | --- | --- | --- | --- | --- |
| Ds | DRAP Oases | 23 495 | 20 736 | 2 052 | 1 940 | 112 | 94.5 | 5.5 |
|  | DRAP Trinity | 39 516 | 30 281 | 935 | 806 | 129 | 86.2 | 13.8 |


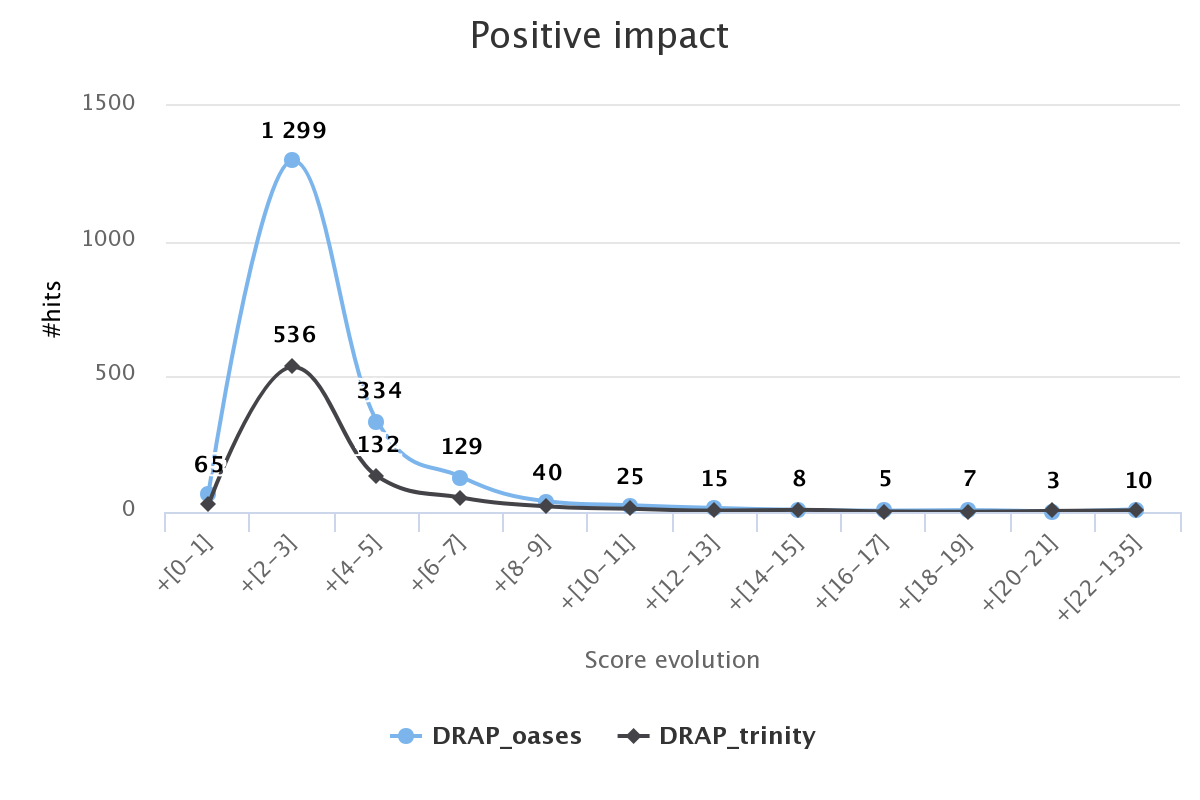
The distribution of the BLAT score evolution for the DRAP contig sets are represented in the following charts.


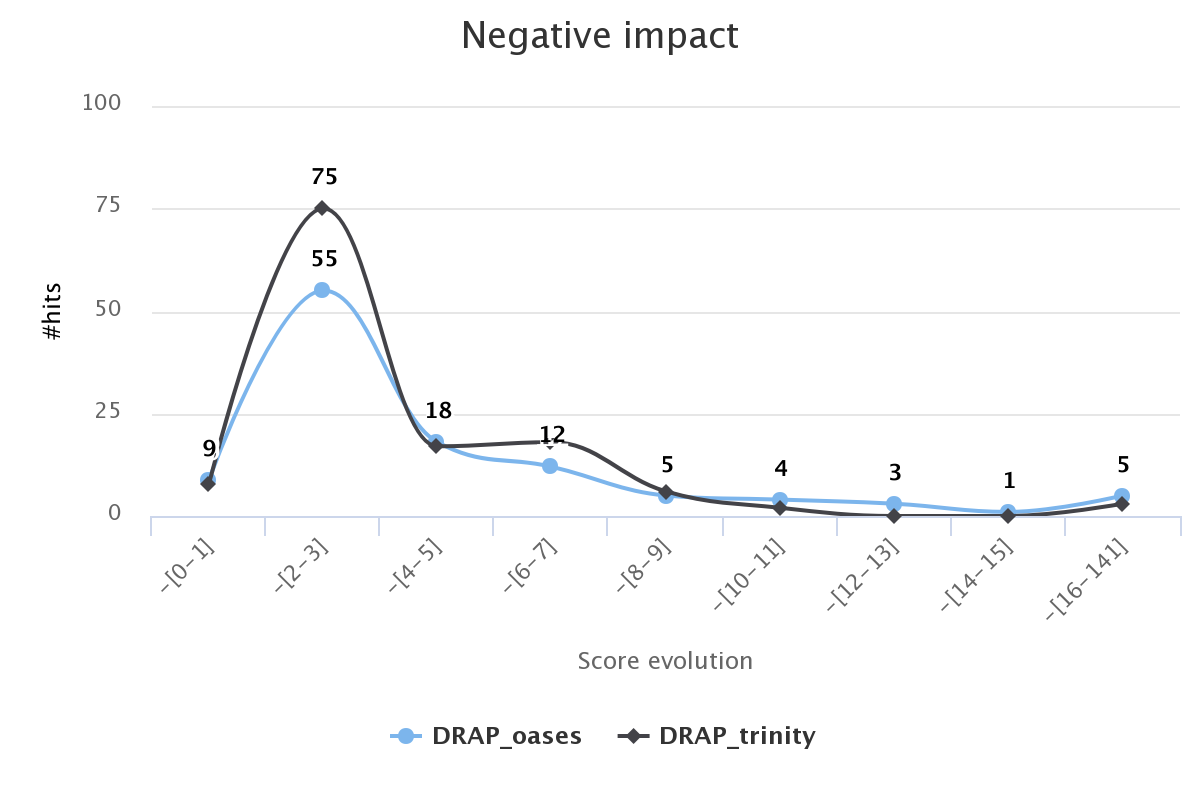

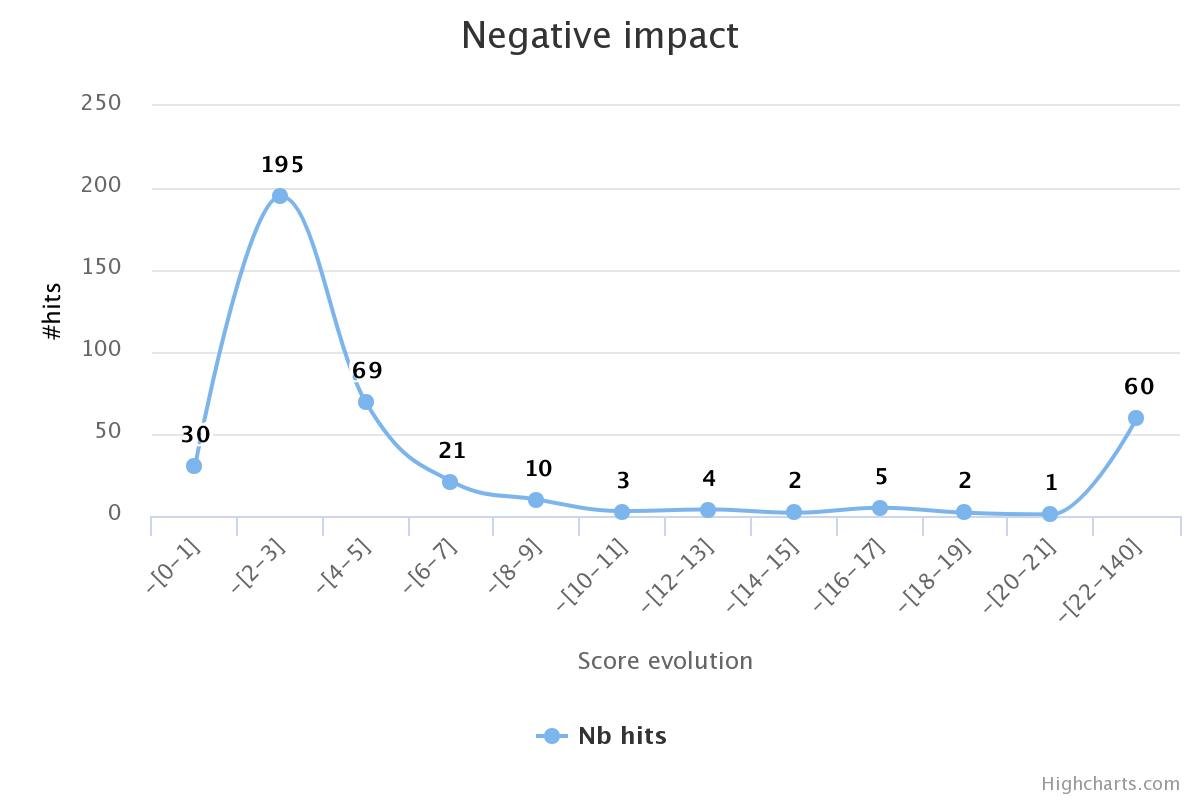
Most of the BLAT scores have increased or decreased of 10 points. Large score drops (-94 and -140 for DRAP Oases and -24 for DRAP Trinity) are due to deletions hosted by a weak majority of the reads. As a negative control, the contig correction step has been applied to the *Danio rerio* reference transcriptome using the simulated dataset (Ds). The reference proteins have been aligned to the reference transcriptome before and after correction. The number of hits between a reference protein and a reference transcript impacted by the correction step are presented hereunder.

| **Dataset** | **Target** | **Nb transcripts** | **Nb transcripts with hit** | **Nb hits with impacted score** | **Nb hits positively impacted** | **Nb hits negatively impacted** | **Positive impact (%)** | **Negative impact (%)** |
| --- | --- | --- | --- | --- | --- | --- | --- | --- |
| Ds | Reference transcripts | 50 160 | 49 946 | 37 | 2 | 35 | 5.4 | 94.6 |

With only 37 transcripts impacted, we can conclude that the correction step has close to no effect when the reads and transcripts contain the same information.

**RunDrap runtimes.**

All runs were performed with the Docker image *sigenae/drap1.9* on a computer with 64G of RAM and 8 CPU's. The assembled dataset is the multi sample Dr dataset. RunDrap was launched with the following command lines on data previously trimmed and normalized.

runDrap --no-trim --no-norm --dbg-mem 60 \
 --outdir SRR10480XX_oases \
 --R1 SRR1048059_1.trim.norm.fq.gz SRR1048060_1.trim.norm.fq.gz \
 SRR1048061_1.trim.norm.fq.gz SRR1048062_1.trim.norm.fq.gz \
 SRR1048063_1.trim.norm.fq.gz \
 --R2 SRR1048059_2.trim.norm.fq.gz SRR1048060_2.trim.norm.fq.gz \
 SRR1048061_2.trim.norm.fq.gz SRR1048062_2.trim.norm.fq.gz \
 SRR1048063_2.trim.norm.fq.gz

runDrap --no-trim --no-norm --dbg-mem 60 \
 -outdir SRR10480XX_trinity --dbg trinity \
 --R1 SRR1048059_1.trim.norm.fq.gz SRR1048060_1.trim.norm.fq.gz \
 SRR1048061_1.trim.norm.fq.gz SRR1048062_1.trim.norm.fq.gz \
 SRR1048063_1.trim.norm.fq.gz \
 --R2 SRR1048059_2.trim.norm.fq.gz SRR1048060_2.trim.norm.fq.gz \
 SRR1048061_2.trim.norm.fq.gz SRR1048062_2.trim.norm.fq.gz \
 SRR1048063_2.trim.norm.fq.gz

As shown in charts below, DRAP requires less than twice the time of a raw assembly and does not exceed the memory required by Oases or Trinity. The longer post-process step after a Trinity assembly is due to the assembler directory cleaning time.


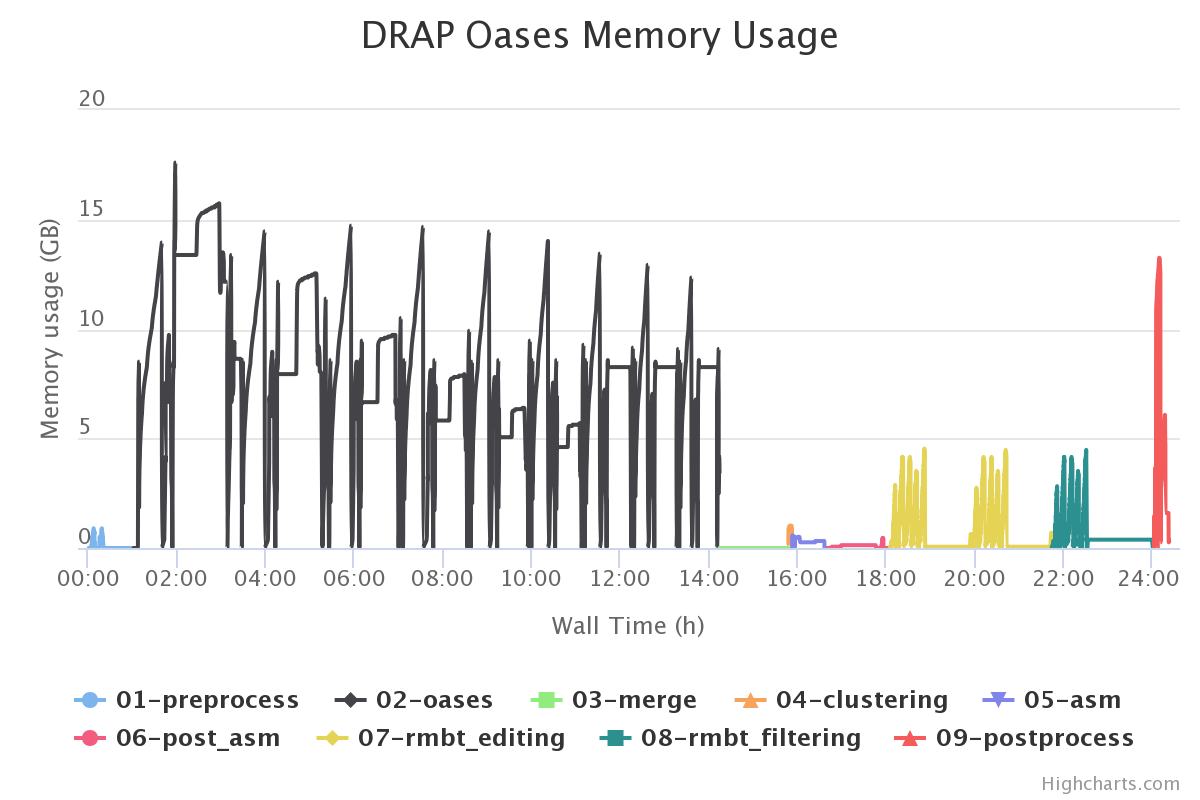


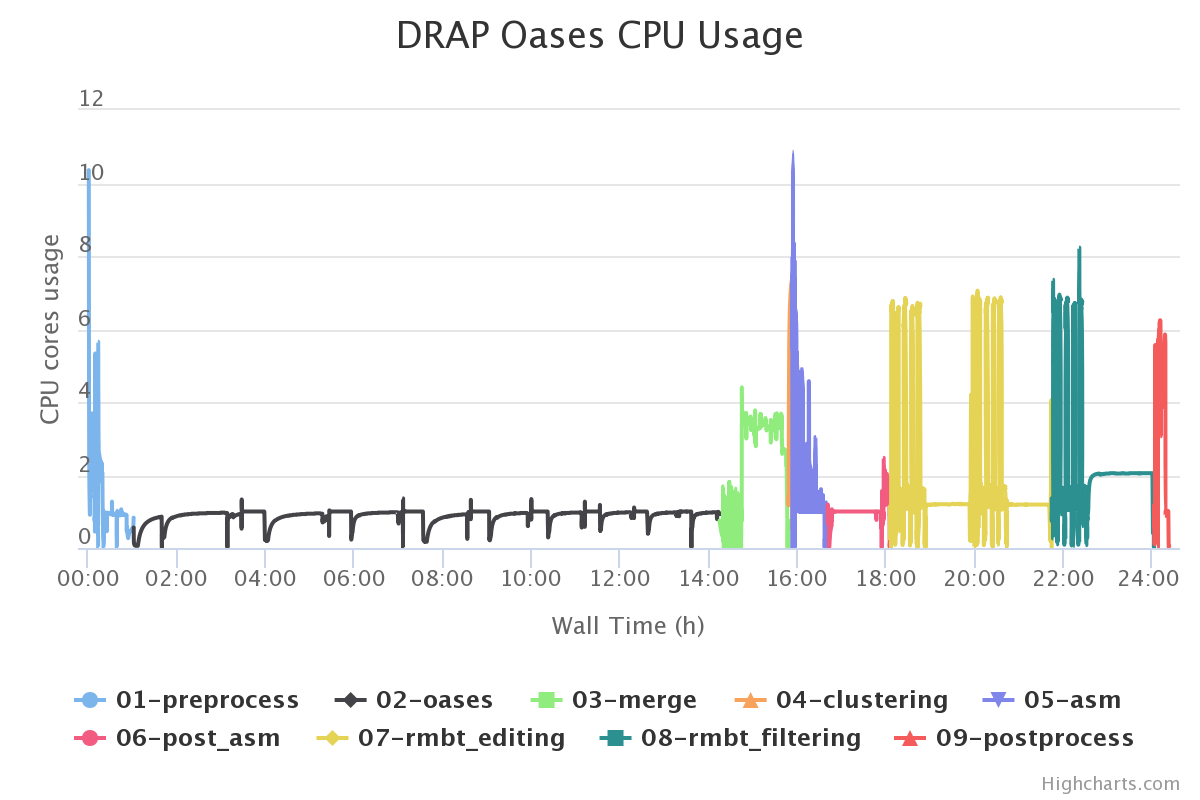

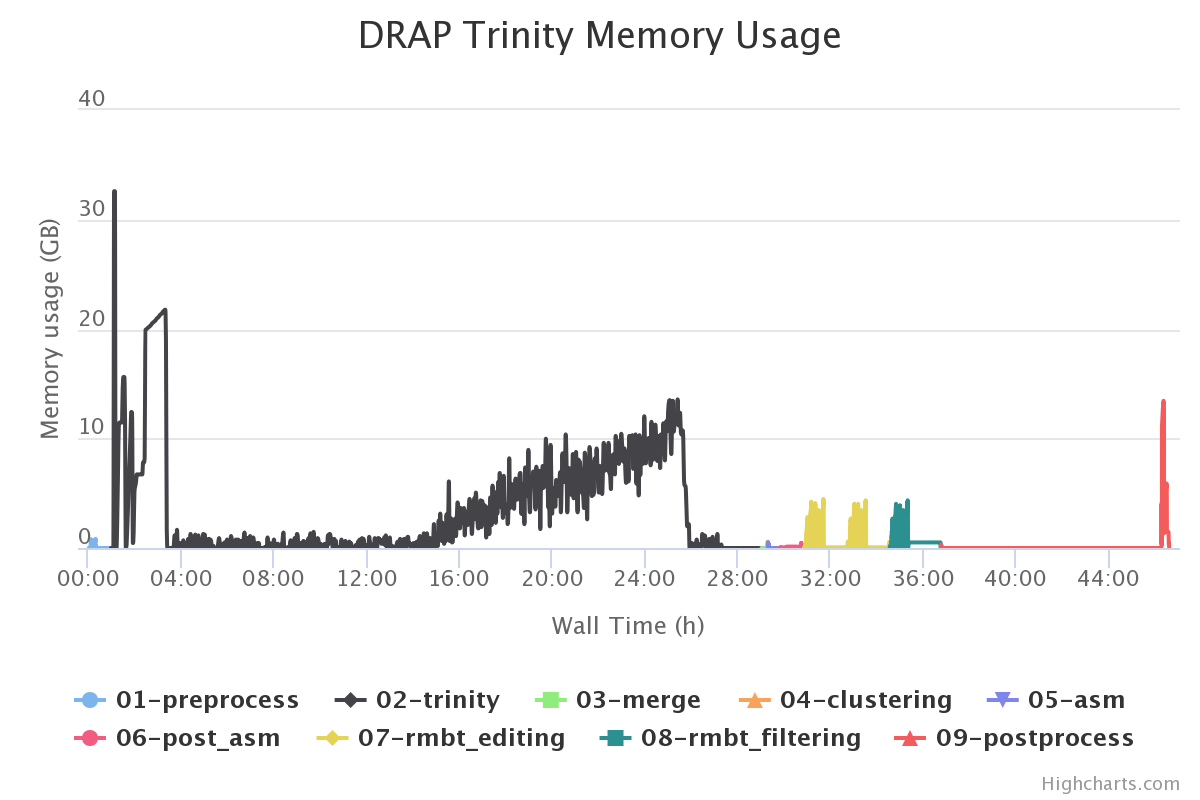


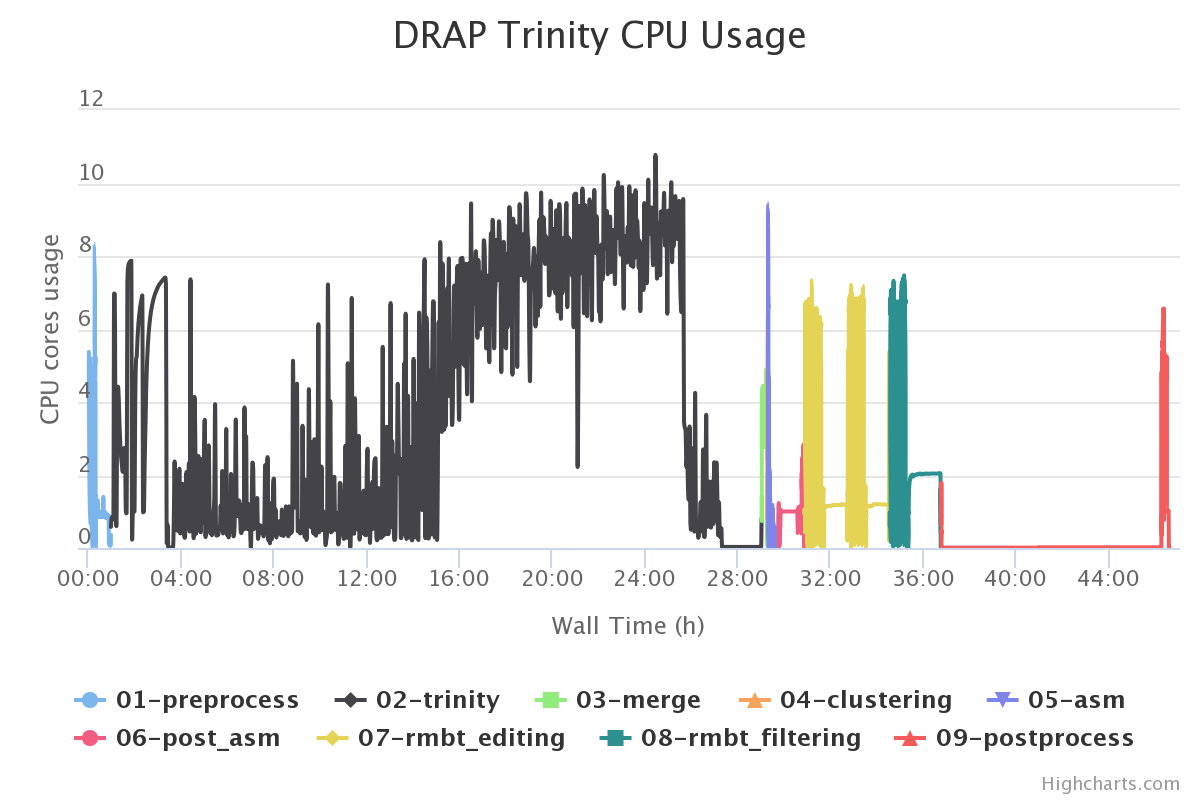

Supplement: Data S1 [file peerj-05-2988-s001.docx]
