# Supplementary material for: Compacting and correcting Trinity and Oases RNA-Seq de novo assemblies
Source: PeerJ. 2017 Feb 16;5:e2988. doi: 10.7717/peerj.2988 (PMC5316280; doi:10.7717/peerj.2988)
Supplement: Table S1 [file peerj-05-2988-s002.docx]

| **Step** | **Package** | **Version** | **Default command** |
| --- | --- | --- | --- |
| Preprocess | trim_galore | 06.05.2015 Cutadapt v0.4.0 | trim_galore --gzip --no_report_file --length %d --output_dir <outdir> --quality 10 \ --stringency 3 --paired <R1.fq> <R2.fq> |
|  | fastq_illumina_filter | 0.1 | fastq_illumina_filter -N |
|  | Khmer | 2.0 | normalize-by-median.py -M %s –paired --output - - |
|  | Trinity | 2.2.0 | insilico_read_normalization.pl --seqType fq --JM %dG --CPU %d --output tmp_norm \ --max_cov 50 --pairs_together --PARALLEL_STATS --left <R1.fq> --right <R2.fq> |
| DBG assembly | Trinity | 2.2.0 | Trinity --no_cleanup --seqType fq --max_memory %dG --bflyHeapSpaceMax 4G --CPU %d \ --output <outdir> --no_normalize_reads --run_as_paired --single %s/Sequences |
|  | Velvet | 1.2.07 | velveth <outdir> 27 -shortPaired -fastq - -noHash  velveth <outdir> <kmer> -strand_specific -reuse_Sequences  velvetg <outdir> -read_trkg yes -min_contig_lgth 100 -cov_cutoff 4 |
|  | Oases | 0.2.06 | oases <outdir> -cov_cutoff 4 |
| DBG cleaning | seqclean | x86_64_20110222 | seqclean <input.fa> -o <output.fa>  cdbyank -l <input.cidx> |
|  | NCBI tools | 6.1 (Dec 2012) | vecscreen -i <input.fa> -d <database.fa> -f 3 |
| Inclusion removing | cd-hit | 4.6 (Feb 10 2015) | cd-hit-est -i <input.fa> -o <outdir> -M 0 -d 0 -c 0.98  cd-hit -i <input.fa> -o <outdir> -M 0 -d 0 -c 0.90 -g 1 |
| OLC assembly | tgicl | 2.1 | tgicl -F <input.fa> -l 60 -p 96 -s 100000 |
| Cleaning | TransDecoder | 2.0.1 | TransDecoder.LongOrfs -t <input.fa>  TransDecoder.Predict -t <input.fa> |
|  | bedtools | 2.22.1 | bedtools merge -c 4 -o distinct -i - |
|  | EMBOSS | 6.4.0.0 | getorf -auto -filter |
| Editing by reads alignment | bwa | 0.7.12-r1039 | bwa [aln\|sampe\|samse] |
|  | STAR | 2.4.0i | STAR --genomeDir <dir> --readFilesIn <R1.fq> <R2.fq> --alignIntronMin 10 \ --alignIntronMax 25000 --outFilterMultimapNmax 10000 |
|  | samtools | 1.1 | samtools [mpileup\|sort\|index\|flagstat\|view\|merge\|faidx] |
| Filtering by reads alignment | express | 1.5.1 | express --no-update-check --no-bias-correct --logtostderr --output-dir <outdir> \ <reference.fa> <input.bam> |
| Scoring | TransRate | 1.0.1-linux-x86_64 | transrate --assembly=<input.fa> --left=<R1.fq> --right=<R2.fq> --threads=%d \ --output=<outdir> |
| Reference alignment | Exonerate | 2.2.0 | exonerate --percent 50 --showalignment no --showvulgar no <queries.fa> <targets.fa> |
|  | Blat | BlatSuite v34 | blat <targets.fa> <queries.fa> -noHead <output.psl> |
