# Supplementary material for: Compacting and correcting Trinity and Oases RNA-Seq de novo assemblies
Source: PeerJ. 2017 Feb 16;5:e2988. doi: 10.7717/peerj.2988 (PMC5316280; doi:10.7717/peerj.2988)
Supplement: Table S2 [file peerj-05-2988-s003.docx]

| **Dataset** | **Assembler** | **Sum(nt)** | **Insertion** |  | **Deletion** |  | **Substitution** |  |
| --- | --- | --- | --- | --- | --- | --- | --- | --- |
|  |  |  | **Cases** | **Ratio*** | **Cases** | **Ratio*** | **Cases** | **Ratio*** |
| At | Oases | 843 329 264 | 2075 | 1.75 | 2545 | 3.14 | 159438 | 3.99 |
|  | DRAP Oases | **56 122 047** | **79** |  | **54** |  | **2656** |  |
|  | Trinity | 130 969 737 | 791 | 5.27 | 548 | 4.05 | 32656 | 4.22 |
|  | DRAP Trinity | **80 258 659** | **92** |  | **83** |  | **4742** |  |
| Bt | Oases | 269 085 141 | 268 | 2.26 | 552 | 4.26 | 16378 | 4.22 |
|  | DRAP Oases | **47 727 730** | **21** |  | **23** |  | **689** |  |
|  | Trinity | 90 989 611 | 212 | 7.19 | 194 | 7.68 | 9533 | 5.62 |
|  | DRAP Trinity | **64 809 448** | **21** |  | **18** |  | **1208** |  |
| Dm | Oases | 232 776 717 | 789 | 2.77 | 997 | 4.84 | 43302 | 6.49 |
|  | DRAP Oases | **29 372 261** | **36** |  | **26** |  | **842** |  |
|  | Trinity | 57 209 890 | 210 | 4.71 | 299 | 7.21 | 15403 | 7.06 |
|  | DRAP Trinity | **37 249 612** | **29** |  | **27** |  | **1421** |  |
| Dr | Oases | 1 059 904 844 | 3118 | 0.49 | 5613 | 1.02 | 157355 | 0.95 |
|  | DRAP Oases | **82 268 872** | **495** |  | **429** |  | **12920** |  |
|  | Trinity | 96 279 046 | 2847 | 10.04 | 1721 | 5.81 | 66819 | 6.11 |
|  | DRAP Trinity | **68 900 396** | **203** |  | **212** |  | **7831** |  |
| Ds | Oases | 280 469 694 | 447 | 4.41 | 715 | 5.99 | 9748 | 2.75 |
|  | DRAP Oases | **46 994 928** | **17** |  | **20** |  | **595** |  |
|  | Trinity | 58 571 859 | 146 | 6.43 | 96 | 3.38 | 6917 | 5.65 |
|  | DRAP Trinity | **51 580 407** | **20** |  | **25** |  | **1079** |  |
| Hs | Oases | 132 681 065 | 214 | 18.64 | 308 | 26.83 | 10616 | 10.55 |
|  | DRAP Oases | **34 670 862** | **3** |  | **3** |  | **263** |  |
|  | Trinity | 47 639 190 | 80 | 12.95 | 67 | 27.11 | 4832 | 14.17 |
|  | DRAP Trinity | **38 557 758** | **5** |  | **2** |  | **276** |  |

(*) ratio between raw and DRAP assembly correction rates
Bold values are “best in class” values between raw and DRAP assemblies
